# GLP-1 Receptor Agonists in Diabetes and Obesity: A Case Report and Review of Bowel Obstruction Risks and Management

**DOI:** 10.7759/cureus.81891

**Published:** 2025-04-08

**Authors:** Matthew Jones, James J Cappola

**Affiliations:** 1 Internal Medicine, Campbell University School of Osteopathic Medicine, Buies Creek, USA

**Keywords:** glp-1 agonist, glp-1 ras, sbo, semaglutide, small-bowel obstruction

## Abstract

Glucagon-like peptide-1 receptor agonists (GLP-1 RAs) are commonly used for managing type 2 diabetes and obesity. These drugs improve glycemic control and promote weight loss, but they can cause gastrointestinal side effects such as gastroparesis and bowel obstruction, particularly in patients with preexisting GI motility issues. A 55-year-old woman with obesity, type 2 diabetes, and a history of small bowel obstruction developed severe abdominal pain and nausea after starting semaglutide. She was diagnosed with small bowel obstruction and treated with nasogastric decompression and bowel rest. Her condition resolved, but the potential risks of continuing GLP-1 RA therapy were reassessed. GLP-1 RAs reduce gut motility by slowing gastric emptying and altering the migrating motor complex (MMC), increasing the risk of conditions like gastroparesis and bowel obstruction. The patient’s prior GI issues and GLP-1 therapy likely contributed to her obstruction. GLP-1 RAs have been linked to bowel obstruction, particularly in those with prior GI motility problems. Clinicians should monitor for symptoms in such patients and weigh the benefits of improved glycemic control against the risk of GI complications. In some cases, discontinuing GLP-1 RA therapy may be appropriate. This case highlights the need for caution when prescribing GLP-1 RAs to patients with a history of gastrointestinal issues. Personalized treatment approaches are essential to balance therapeutic benefits with risks. Further research is needed to understand the mechanisms of GLP-1 RA-induced bowel obstruction.

## Introduction

Glucagon-like peptide-1 (GLP-1) is an incretin hormone released from the intestine after the ingestion of glucose or nutrients. GLP-1 receptor agonists (GLP-1 RAs) are approved for the treatment of type 2 diabetes and obesity and have gained widespread use due to their effectiveness in reducing both A1c levels and body weight. As of recent estimates, over 4 million prescriptions for GLP-1 RAs were written from 2018 to 2024 in the United States [1]. GLP-1 RAs work by stimulating insulin secretion from pancreatic β-cells, improving glycemic control [2]. They also promote β-cell proliferation, suppress glucagon release, and slow gastric emptying. However, their effects on gastrointestinal motility may lead to adverse events, including gastroparesis and pancreatitis [3]. Gastrointestinal side effects are among the most commonly reported adverse events with GLP-1 RAs. Studies indicate that nausea occurs in approximately 15-50% of users, vomiting in 5-20%, and constipation in 4-12% [4]. These effects are dose-dependent and may influence long-term adherence to therapy. Bowel obstruction is a medical emergency that, if left untreated, can lead to severe complications, including sepsis, aspiration, bowel perforation, and death [5]. It occurs due to a blockage within the intestinal lumen and presents with symptoms such as abdominal pain, vomiting, constipation, nausea, and abdominal distention.

## Case presentation

**Figure 1:**
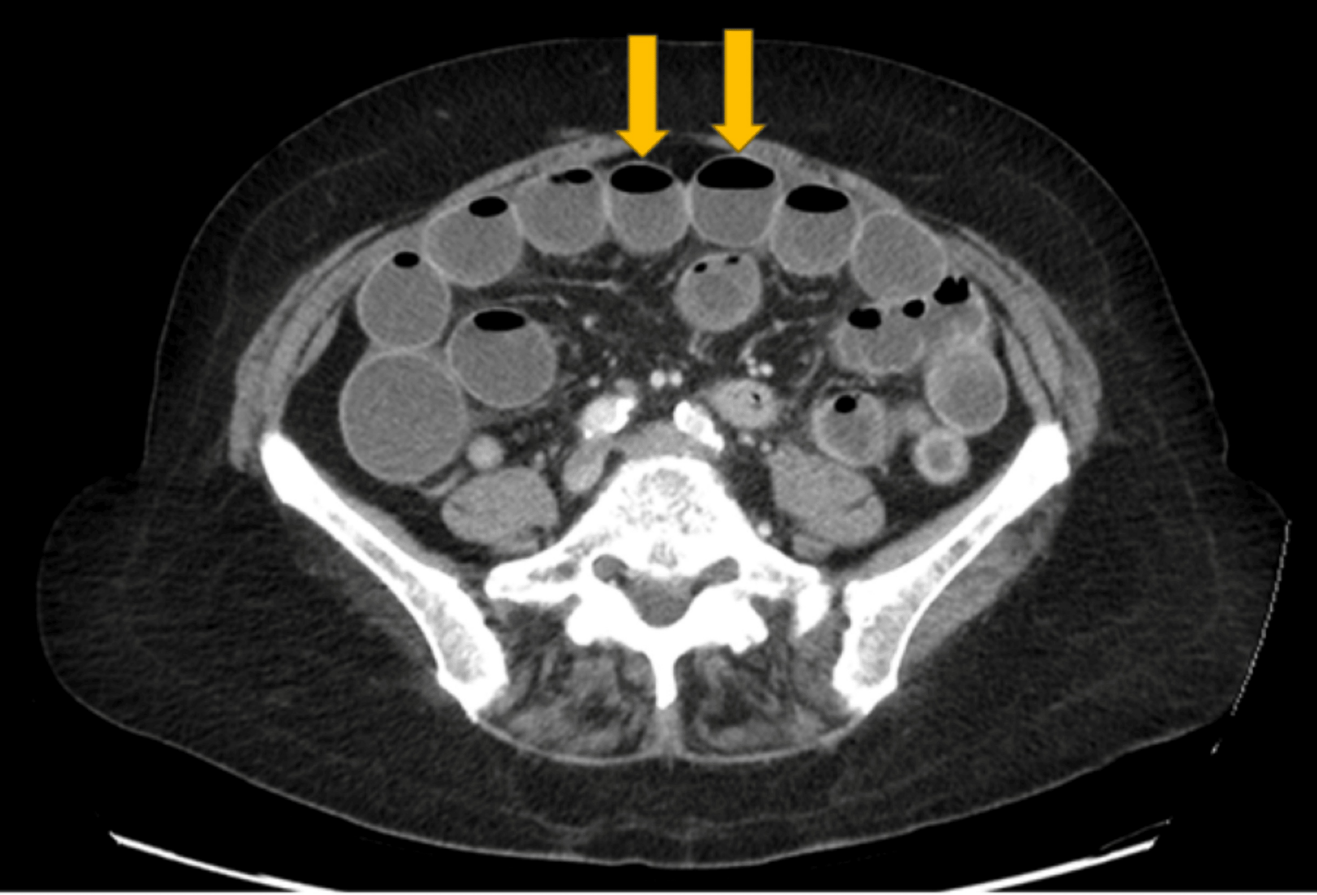
Example of small bowel obstruction on CT

We present a clinical vignette based on a 55-year-old woman with a history of obesity, type 2 diabetes mellitus, and a previous small bowel obstruction treated medically one year earlier. She was treated at a rural hospital in North Carolina affiliated with the Harnett Health Internal Medicine Residency program. She presented to the emergency department with severe abdominal pain, nausea, and vomiting. Six weeks prior to her current presentation, she was prescribed the GLP-1 RA semaglutide (Ozempic) at a dose of 0.5 mg weekly for diabetes control and weight management. Radiologic evaluation confirmed multiple dilated small bowel loops and air-fluid levels, confirming a small bowel obstruction (Figure 1).

Her treatment included nasogastric tube decompression, intravenous fluids, and bowel rest. The obstruction resolved completely within four days. Her care team advised against restarting her GLP-1 RA and recommended avoiding a dipeptidyl peptidase-4 inhibitor (DPP-4i) for diabetes management. Although her small bowel obstruction resolved with non-surgical treatment, the patient and her physician carefully reassessed the risks and benefits of continuing GLP-1 therapy. While her diabetes management has improved, the risk of recurrent bowel obstruction remains a concern, especially given her history of prior obstruction.

## Discussion

The pathophysiology of GLP-1 RA-induced bowel obstruction is multifactorial. GLP-1 receptors are present throughout the GI tract, including the stomach, small intestine, and colon [6]. Their activation reduces peristalsis, slows gastric emptying, and alters the migrating motor complex (MMC), which regulates gut motility during fasting periods. This delayed transit time through the stomach and small intestine increases the risk of conditions such as gastroparesis and bowel obstruction. While the delayed gastric emptying and altered MMC are primarily secondary consequences of GLP-1 RA activation, they indirectly contribute to delayed small bowel transit. Additionally, GLP-1 receptors are expressed in the small intestine, where their direct activation can further inhibit motility. This dual mechanism (direct and secondary) likely contributes to the overall motility dysfunction observed with GLP-1 RA therapy, which can result in bowel obstruction, particularly in individuals with preexisting motility issues. GLP-1 RAs may also inhibit the sympathetic nervous system by activating the GLP-1 receptor on the greater and lesser splanchnic nerves [7], as well as the sympathetic nerve branch of Auerbach’s plexus. This results in an inhibitory effect that slows intestinal peristalsis, delays gastric emptying, and increases duodenal-small bowel emptying time, further impairing motility. 

Although more commonly associated with gastroparesis, evidence suggests that GLP-1 RAs can also delay small bowel transit, leading to small bowel obstruction in susceptible individuals. In the patient described, her GLP-1 RA therapy may have contributed to her recurrent small bowel obstruction, particularly given her history of bowel obstruction. However, other potential causes, such as adhesions from prior obstruction, other medications, or underlying bowel disease, were considered but deemed unlikely. The patient’s medical history did not reveal significant adhesions, she was not on other drugs known to induce bowel obstruction, and no underlying bowel disease was identified. Furthermore, the timing of the obstruction, which correlated with the initiation of GLP-1 RA therapy, suggests a causal relationship.

A pharmacovigilance study using VigiBase found 501,244 adverse events reported with diabetes drugs between 2007 and 2018, with 698 cases involving intestinal obstruction [8]. Of those, 333 (47.8%) cases involved a GLP-1 RA with a reporting odds ratio (ROR) of 4.52 (95% CI: 3.87-5.28), and 398 (57.1%) involved a DPP-4 inhibitor with an ROR of 8.66 (95% CI: 7.27-10.32). Given the potential for small bowel obstruction, it is important to consider individualized care in patients prescribed GLP-1 RAs, particularly in those with a history of GI motility disorders. Clinicians should remain vigilant for symptoms of GI distress, such as nausea, vomiting, or abdominal pain, and adjust treatment accordingly. For patients at higher risk, careful monitoring may be warranted, and alternative therapies could be considered. The development and implementation of clear, evidence-based prescribing guidelines for GLP-1 RAs are essential to aid clinicians in identifying high-risk patients and tailoring therapy appropriately. These guidelines should include recommendations for baseline risk assessment, monitoring parameters, and stepwise management strategies in the event of adverse GI effects.

## Conclusions

We present a case of a patient with a history of bowel obstruction who was prescribed a GLP-1 RA six weeks prior to her second presentation with bowel obstruction. After discontinuing the GLP-1 RA and administering medical therapy, including bowel rest, nasogastric decompression, and intravenous fluids, her condition resolved without the need for surgery. This case highlights the potential risk of recurrent bowel obstruction in patients on GLP-1 RAs, suggesting that in certain cases, permanent cessation of therapy may be warranted. However, decisions regarding discontinuation should be individualized based on patient-specific risk factors, including prior gastrointestinal issues and response to therapy.

Large post-marketing studies indicate that the risk of bowel obstruction is approximately four times greater in patients using GLP-1 RAs. This risk may be more pronounced in individuals with preexisting gastrointestinal motility disorders, but it remains essential to assess this risk for all patients on GLP-1 RA therapy. Clinicians should carefully weigh the benefits of these medications for glycemic control and weight loss against the potential risks of bowel obstruction, pancreatitis, and other complications. Close monitoring for symptoms of bowel obstruction, especially in patients with a history of GI motility disorders, is essential. Additionally, clinicians should review concomitant medications such as opioids, anticholinergics, or other agents that may reduce gastrointestinal motility, which could exacerbate these risks. While permanent discontinuation may be appropriate in some cases, other strategies, such as dose reduction or switching to an alternative GLP-1 RA with a different pharmacokinetic profile, may help preserve the metabolic benefits of therapy while mitigating the risk of bowel obstruction. Further research is necessary to better understand the mechanisms of GLP-1 RA-induced bowel obstruction and inform clinical guidelines for managing high-risk patients.
